# Knowledge Domain and Emerging Trends of Social Vulnerability Research: A Bibliometric Analysis (1991–2021)

**DOI:** 10.3390/ijerph19148342

**Published:** 2022-07-08

**Authors:** Hua Li, Weijun Wang

**Affiliations:** 1 College of Economics, Northwest Normal University, Lanzhou 730070, China; lihua0529@nwnu.edu.cn; 2 College of Tourism, Northwest Normal University, Lanzhou 730070, China

**Keywords:** social vulnerability, CiteSpace, knowledge mapping, research hotspot, development trend

## Abstract

Carrying out social vulnerability research has become an important way to understand the sustainable development of resources, the environment, populations, and societies. Clarifying the research context and development trend of social vulnerability is of great significance to the follow-up theoretical research on and practical exploration of sustainable social development. With the help of a CiteSpace knowledge map analysis, this study reveals the research hotspots and their evolution in different periods and puts forward the key problems in and future direction of social vulnerability research in the future. This study found that the number of articles on social vulnerability research showed a steady upward trend and that the research experienced roughly three stages: an embryonic stage, a development stage, and a stable stage. The United States, Brazil, the United Kingdom, and China accounted for the majority of the research, but the intensity of cooperation between them is still weak. Vulnerability assessment and risk assessment in the context of policy and environmental change are hot topics in the current research. In the future, it is necessary to focus on the comprehensive research on the integrated and cross-scale research on social vulnerability, research on its occurrence and evolution, and on the dynamic monitoring as well as optimal regulation of social vulnerability under multiple pressures.

## 1. Introduction

In recent years, the dual effects of natural and human factors, such as climate change, rapid urbanization, and environmental pollution, have led to the frequent occurrence of various natural disasters and public security events around the world as well as the increasing vulnerability of social systems, which seriously threaten social sustainable development [1,2,3]. According to the fifth assessment report of the Intergovernmental Panel on Climate Change (IPCC) and the global risk report, the risks faced by human societies will continue to increase in the future, and the risks will become more systematic and complex [4,5]. How to resist the impacts of risks, improve the system response ability, and reduce the vulnerability of social systems has become a major practical problem faced by scholars and governments [6,7,8,9]. At present, vulnerability research has become a hot topic, concerning many international scientific programs and institutions (the IPCC, UNISDR, IHDP, etc.) [10]. Identifying vulnerable groups and regions in addition to exploring vulnerability mechanisms and adaptation strategies not only helps to reduce the adverse impact of environmental changes and human activities on the social system but also has become an important way of quantifying the sustainable development of resources, the environment, populations, and societies [1,11,12,13].

As one of the dimensions of vulnerability, social vulnerability refers to when a social system is exposed to a disaster or environmental risk impact or disturbance, in which it is negatively affected or damaged due to its own sensitivity characteristics and lack of ability to deal with adverse disturbances [2,6,7]. At present, many scholars have carried out extensive studies on social vulnerability from the perspectives of climate change, natural disasters, urbanization, tourism development, infectious diseases, environmental pollution, and public security. The research results cover multidisciplinary fields, such as disaster science, geography, and sociology [3,13,14,15]. The content involves the construction of index systems [1,16,17] and measurements [2,3,18,19] of social vulnerability as well as the spatio-temporal differentiation characteristics [2,3,16] and identification of vulnerable groups [20,21,22]. A few studies have explored the influencing factors, formation mechanisms [2,21,23], and adaptation strategies [24,25,26] of social vulnerability. The research methods mostly adopt a comprehensive index method, AHP cluster analysis method, principal component analysis method, function model method, BP neural network model, set pair analysis method, and spatial multicriteria evaluation method [18,21,27]. Overall, the current research results on social vulnerability are relatively abundant, but most of them take typical case areas as examples to carry out a traditional descriptive analysis or statistical analysis on relevant topics in a specific background. Visual analyses and summaries of the evolution process and development trend of the knowledge in social vulnerability research are relatively lacking.

Analyses of the knowledge structure and evolution of research topics in specific areas have been the focus of information science. These analyses help interested researchers to enrich their understanding of the knowledge domain of a particular research topic. Under the background of complex changes in the global environment and the realistic requirement of the SDGs, conducting social vulnerability research not only helps to reduce the adverse impacts of environmental changes on social systems but has also become an important way to promote the sustainable development of national or regional resources, the environment, populations, and societies, and it is also a key link in building resilient societies, cities, and villages. Therefore, clarifying the research context and development trend of social vulnerability can provide researchers with an effective and rapid research network as a comprehensive and overall picture of knowledge development so as to provide basic inspiration for proposing new research questions and constructing new perspectives. However, up to now, the existing surveys have focused on the discussion of social vulnerability in specific regions and topics as well as studies within a limited period, paying little attention to the objective and quantitative description of the knowledge field of social vulnerability and the overview of research trends.

In recent years, with the increasing maturity of the scientific knowledge mapping method, it has been widely used to explore the internal structures, discipline characteristics, and research frontiers of discipline systems. CiteSpace is a very effective analysis software for the scientific knowledge mapping method. It can quickly and comprehensively understand the hot topics and research directions of a research field by generating and analyzing a scientific knowledge map of keywords, topic categories, authors, institutions, and countries in a specific field and is conducive to monitoring the development and trend of a discipline [28].

Based on this, our analysis included 2710 studies collected in the *Web of Science* (*WoS*) database to carry out a visual analysis of social vulnerability research and summarized the research process, research hotspots, and trends in the field of social vulnerability. Furthermore, we propose the research topics that need to be focused on in the future. On the one hand, this research can enrich the research scope of social vulnerability, help monitor the research development of this topic, investigate its research changes, and predict future research trends. On the other hand, this study provides an overall overview of the knowledge structure for researchers, especially students and novices in this field, which is of great significance for theoretical research on and the practical exploration of social vulnerability.

## 2. Methodology

### 2.1. Data Collection

The data used in this study for bibliometric analysis are from the *Science Citation Index Expanded* (*SCIE*), *Social Sciences Citation Index* (*SSCI*), and *Arts and Humanities Citation Index* (*A&HCI*) databases in *Web of Science* (*WOS*). Before performing a topic search in *WOS*, we reviewed the previous literature and made several attempts to identify key terms. The “vulnerability” or both “vulnerability” and “society” search subject words indicate that the research focuses on the vulnerability of natural and ecological systems or the impact of the vulnerability of natural resources, ecosystems, and biological communities on social development. Finally, the subject words were limited to “social vulnerability”, the study types were limited to “article” and “review”, the retrieval period was set from 1990 to 2021, and the retrieval time was 1 February 2022. The search results were manually screened, and invalid data, such as prefaces, conference drafts and reports, personal academic profiles, book reviews, and irrelevant items, were deleted. Finally, a total of 2710 results were obtained. Although we excluded some relevant papers from the selection of subject search words, we believe that the sample analyzed here is large enough to enable a systematic discussion of our findings.

### 2.2. CiteSpace

CiteSpace is a data-mining and visualization software with a powerful citation analysis function. It can construct bibliometric networks in different time periods to identify the development trends and citation hotspots of research topics and detect as well as visualize emergent items and mediating centrality to identify sudden changes and important turning points of research topics [28] so as to visually display the knowledge structure and development context of a topic for researchers. This study uses CiteSpace 5.7.R2 software to produce a co-occurrence visualization map of countries/regions, institutions, keywords, and cited publications. These key items perform in the form of ring nodes; the color and size of the rings correspond to the year and frequency of the items, respectively. The greater the co-occurrence frequency of the items, the larger the node. The thickness of the connection can indicate the closeness and distance between the items. Then, according to the changing trends of items (rather than just the frequency), the bursts and duration of an item can be analyzed so as to determine the frontier issues and important turning points of research topics in different periods. 

## 3. Results Analysis 

### 3.1. Publication Analysis

**Figure 1:**
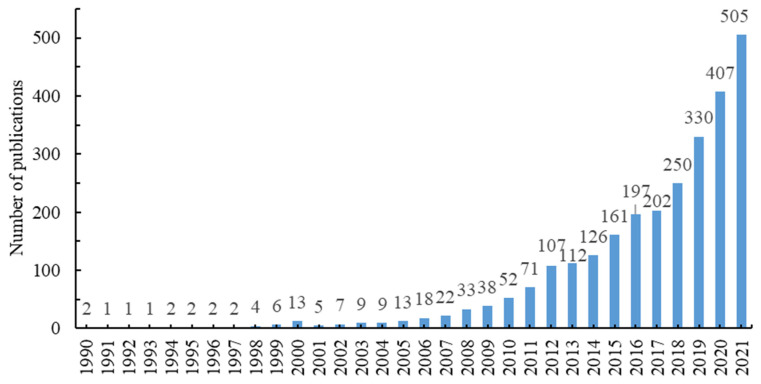
Number of publications on social vulnerability research from 1990 to 2021.

As shown in Figure 1, from 1990 to 2021, the amount of literature on social vulnerability research showed a steady upward trend, with an average annual growth rate of 25.5%, but there were great differences in different years. According to the amount of literature published and its annual growth rate, the research process of social vulnerability can be divided into three stages. The first stage is the embryonic period (1990–1999). During this period, research on social vulnerability began to emerge, and few scholars paid attention to it. A total of 23 papers were published in these 10 years, with an average annual growth rate of about 22.2%. The second stage is the development period (2000–2012). In the context of intensified environmental changes, such as climate change and resource depletion, many research institutions put forward topics such as “vulnerability” and “adaptability”, which led to a significant increase in the literature on social vulnerability research during this period, and the research topics were constantly diversified and deepened. In these 13 years, 397 papers were published, with an average annual growth rate of about 32.2%. The third stage is stable. During this period, the research on social vulnerability received continuous attention, and the scale of literature increased year by year. There were 2290 articles published in 9 years, but the average annual growth rate (19.2%) showed a downward trend. 

### 3.2. Major Contributions Countries/Regions and Institutions to Social Vulnerability Research 

**Figure 2:**
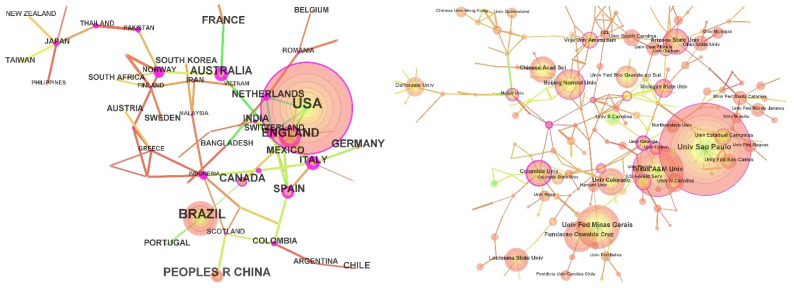
Co-occurrence visualization map of countries (**left**) and institutions (**right**) undertaking social vulnerability research.

Cooperation is an important part of scientific research. Researchers and their research network relationships are the basic elements of research disciplines. A single scientist can rarely provide all of the professional knowledge and resources needed to solve complex research problems [29]. Therefore, we used CiteSpace to generate a network based on an author’s organization and country. This analysis can identify representative countries and key institutions of social vulnerability research, which can provide a basis for relevant scholars to continue their research and find partners. A one-year time slice from 1990 to 2021 was selected for analysis, the most significant links were retained using Pathfinder network pruning, and the top 50 most frequent items were selected from each year. In the co-occurrence network, each node represents a country/region or institution, and the line and thickness between nodes indicate their cooperative relationship and strength, respectively. The range of color indicates the time sequence of the co-occurrence of authors, countries, or institutions; the color change from blue to orange indicates the change from earlier to recent times; the purple circle indicates key research with a centrality higher than 0.1; and the thicker circle indicates a higher centrality. Figure 2 depicts the symbiotic relationship of countries/regions and institutes related to social vulnerability research, scaled and simplified by Pathfinder networks. 

As seen in Figure 2, the collinear network of the main contributing countries is composed of 60 nodes and 74 links. The main contributions of social vulnerability research come from the United States, Brazil, the United Kingdom, and China, accounting for 62.66% of the total number of published papers, indicating that these countries have widely established the discipline system of social vulnerability research. Among them, 924 papers were published in the United States, which is an absolute leader in the study of social vulnerability. It is followed by Brazil, the United Kingdom, and China, with 386, 197, and 191 papers, respectively. It is worth noting that the United States and the United Kingdom carried out research on social vulnerability at almost the same time but that the number of publications in the United States was 4.7 times more than that of the United Kingdom. In addition, Switzerland (0.73), the United Kingdom (0.43), Australia (0.55), Norway (0.65), and Indonesia (0.46) have high centrality and occupy key positions in the regional network of social vulnerability research, indicating that these countries have laid the foundation for research in this field and have far-reaching influence. The number of papers published in China is relatively high, but the centrality is only 0.01, indicating that the influence of published papers on social vulnerability in China is still weak and that relevant studies on social vulnerability should be continuously strengthened in the future. Brazil (9.07), the United Kingdom (9.25), France (7.48), and Canada (6.24) have higher emergent degrees, indicating that these countries have played an important role in the innovation of social vulnerability research.

From the visual network of the co-occurrence of research institutions, 314 research institutions carry out social vulnerability research, which initially formed a stable cooperation network, but the cooperation intensity is weak, and academic exchanges as well as cooperation between them should be strengthened in the future. Among them, the large-scale cooperation network includes the internal cooperation network of Brazil (mainly based in the University of Sao Paulo, Universidade Estadual de Campinas, Universidade Federal de Minas Gerais, Fundacao Oswaldo Cruz, etc.), the internal cooperation network of the United States (mainly based on Texas A&M University, the University of North Carolina, and the U.S. Forest Service), and the transnational cooperation network dominated by institutions such as Beijing Normal University in China, Vrije University Amsterdam in the Netherlands, Arizona State University in the United States, and Durham University in the United Kingdom. It is also found that the study of social vulnerability is mainly concentrated in universities. From the perspective of centrality and emergence, the centrality of Columbia University (0.22) and Arizona State University (0.17) in the United States as well as Vrije University Amsterdam (0.19) in the Netherlands is higher, while the emergence of the University of South Carolina (7.34), the University of Sao Paulo (5.67), and Universidade Federal de Minas Gerais (5.59) in Brazil is higher, and their half-life is more than 10 years. According to the analysis of the centrality, emergence, and half-life of research institutions, it can be seen that the research results of these institutions have high academic value and great influence and have played an important role in social vulnerability research. In the future, we should focus on the research results of these institutions and strive to seek cooperation.

### 3.3. Keyword Co-Occurrence Analysis

Keywords are an author’s summary and refinement of the core content of the article. A keywords co-occurrence visualization map can effectively monitor the research hotspots and internal connections in the research topic knowledge field [30]. A timespan from 1990 to 2021 with a one-year time slice was selected for the analysis, and the selection criteria included the top 50 most frequent items per slice with global pruning carried out using the Pathfinder algorithm. Among them, each node represents a keyword. The larger the node, the higher the keyword frequency and the more connections, indicating that the keywords are cross-studied by multidisciplinary scholars. The thicker the connection, the stronger the connection.

**Figure 3:**
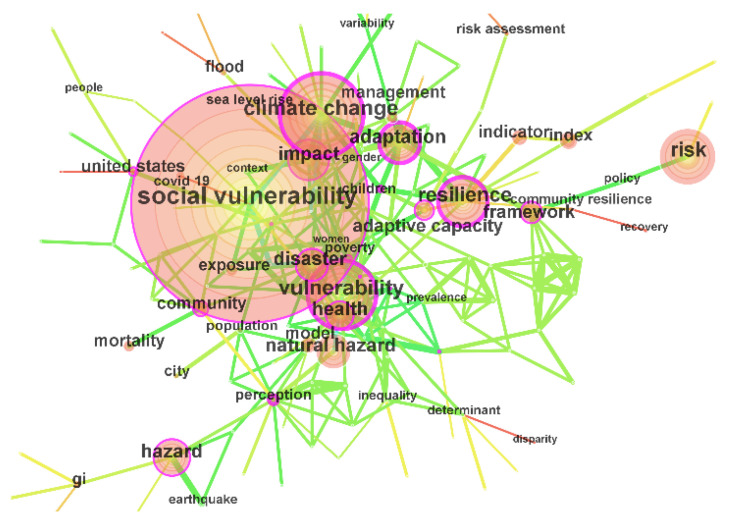
Keyword co-occurrence network of social vulnerability research from 1990 to 2021.

As shown in Figure 3, the keyword co-occurrence visualization map is composed of 144 nodes and 264 links. Since the search subject term is “social vulnerability”, “social vulnerability” has the highest frequency of 1507, followed by “climate change” and “vulnerability”, with frequencies of 572 and 484, respectively. Other high-frequency keywords include “risk” (404), “resilience” (355), “impact” (317), “hazard” (302), and “adaptation” (290). These keywords have been the key topics of social vulnerability research in the past 32 years. In terms of time periods, from 1990 to 2005, there were few keywords on social vulnerability research, and only 24 high-frequency keywords appeared in 16 years, mainly focusing on vulnerability, poverty, disasters, diseases, and adaptability, and the research content was relatively simple. From 2006 to 2021, the number of keywords soared, and 120 high-frequency words appeared in 16 years, including multiple risks, climate change, land use, health, social equality, policy, management, resilience, sustainability, etc. The research on social vulnerability showed a diversified trend.

The purple circle of the node indicates the key points with a node centrality greater than 0.1, which can be used to measure the importance of keywords. As shown in Figure 3, “perception” has the highest centrality (0.32), followed by “climate change” (0.30), “resilience” (0.26), “adaptation” (0.26), “vulnerability” (0.25), and “agriculture” (0.25). It can be seen that social vulnerability research focuses on climate change perception, vulnerability, and resilience.

**Figure 4:**
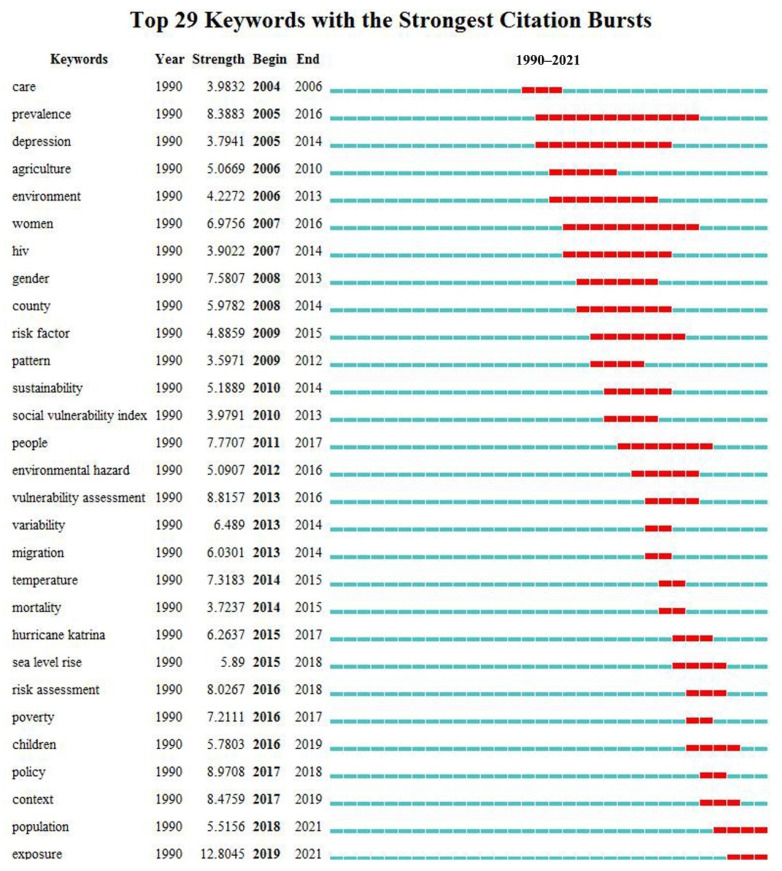
Top 29 keywords with the strongest citation bursts from 1990 to 2021.

Keyword burst detection can identify emerging trends in the research field, indicating that a topic has received special attention from the academic community in a certain period of time [31]. Figure 4 shows 29 emergent words in the field of social vulnerability research from 1990 to 2021. The red range indicates the time period with the largest change in frequency. These keywords were the main interest points of social vulnerability research in the past 32 years. We further screened out the articles that used these keywords with high emergent intensity during emergent time periods and browsed the titles and abstracts of the articles with a high citation frequency, thereby allowing us to preliminarily summarize the topic trends of social vulnerability research in different periods. In general, there were fewer emergent words from 1990 to 2010, but their influence cycle was long. Among them, the keyword with the highest outbreak intensity and the longest impact cycle was “prevalence” (8.39), which experienced a major outbreak from 2005 to 2016. Other keywords with high outbreak intensity were “gender” (7.58), “women” (6.96), “county” (5.98), “sustainability” (5.19), and “agriculture” (5.07). It can be seen that the study of social vulnerability in this period mainly focused on rural vulnerability and gender vulnerability. From 2011 to 2021, the number of emergent words increased rapidly, but the influence cycle was generally short. Among them, the keyword with the highest outbreak intensity was “exposure” (12.80), the keyword with the longest impact cycle was “people” (7.77), and other keywords with high outbreak intensities included “policy” (8.97), “vulnerability assessment” (8.82), “context” (8.48), and “risk assessment” (8.03). It can be seen that vulnerability assessments and risk assessments in the context of policy and the environment are hot topics in this period.

### 3.4. Research Cluster of Social Vulnerability

**Figure 5:**
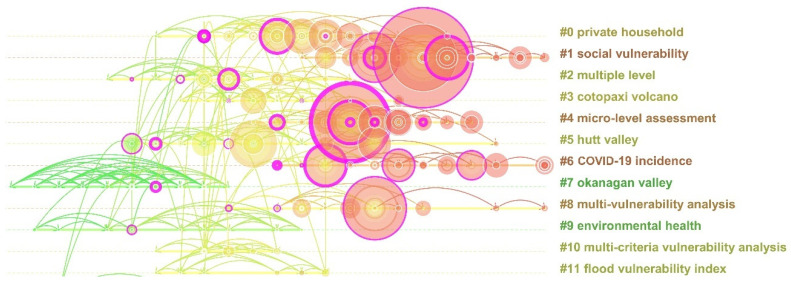
Co-citation timeline visualization of the 11 clusters.

An analysis of the clustering and key nodes in the publication co-citation network can not only reveal the knowledge structure of the research field and the evolution characteristics of the research frontier but also identify the key studies in the evolution process [32]. Based on 87,834 citations in 2710 documents, the 30 items with the highest citation frequencies every year were selected to draw a visual co-citation map (Figure 5), for which the Pathfinder algorithm was used to prune. The network is composed of 309 nodes and 568 connections and generates a total of 33 clusters, of which the modular Q is 0.81, and the average silhouette is 0.44, indicating that the network clustering homogeneity is high, and the clustering results are reasonable. The larger the node, the higher the co-citation frequency of the document, and the changing of the line color indicates the time evolution. The most frequently cited publication in each group is used to describe the characteristics of the group, and the most actively cited publication represents the research frontier in the field. In addition, if the clustering contains a certain number of emerging citations, it is considered to be a new development trend [28]. Table 1 summarizes five key clusters with more than 25 nodes, accounting for about 50% of all of the nodes. The average year represents the typical research period of the literature in the cluster.

The largest cluster (#0) contains 38 pieces of literature, and the silhouette value is 0.81, indicating that the cited publications in this cluster have high consistency. This cluster was named “private household” by LLR and contains 15 citation bursts that focus on the study of the social vulnerability of individuals or families and other groups. The most active citation is Tate: he discussed a method of constructing a social vulnerability index and used the uncertainty analysis method to evaluate and visualize the variability of a hierarchical social vulnerability index [33]. The most frequently cited is Wood (2010), who used an improved social vulnerability index to assess the social vulnerability of a Pacific coast tsunami; this method can help emergency managers to identify community subgroups that are more susceptible to loss and to develop risk reduction strategies that are tailored to local conditions [34]. Fekete (2009) has the highest centrality, which is an important knowledge turning point of cluster #0. Fekete generated a social vulnerability map covering all of the counties in Germany based on demographic variables and verified the families affected by floods in three states through a questionnaire survey. The research shows that the elderly and the poor are indeed more vulnerable to floods [35].

The second largest cluster (#1) contains 31 articles, with a silhouette value of 0.97 and mainly published around 2015. This cluster is named “social vulnerability” by LLR, which focuses on the evaluation of social vulnerability and the analysis of driving factors. The most active citation is Ran. The author used the PRISMA method to systematically evaluate the application of a social vulnerability and resilience framework in low-income and middle-income countries and proposed that more hypothesis-driven research is needed to better understand the mechanisms of vulnerability and resilience that shape disaster preparedness, response, and resilience [1]. The most frequently cited piece of publication is Rufat (2015). Through 67 flood case studies, the author analyzes the driving factors of social vulnerability and believes that demographic characteristics, socioeconomic status, and health are the main driving factors for social vulnerability to flood events. The role of risk perception and coping ability is also prominent but often ignored [36]. The publication with the highest centrality is Armas (2013). The author studied the social vulnerability index (SoVI model) and spatial multi-standard social vulnerability index (SEVI model) using Romanian census data and checked whether they overlapped with known vulnerabilities through exploratory spatial data analysis (ESDA) [37].

The third largest cluster (#2) contains 30 articles, with a silhouette value of 0.73. Most of the research results were published around 2006. This cluster is called “multiple level” and contains seven citation bursts mainly focusing on the vulnerability and adaptability research on multiregional levels or multiple risks. The most active citation is Bisaro. Based on field research, the author discusses the impact of discourse at the international level on climate change adaptation policies at the national and local levels [38]. The most frequently cited publication is Smith (2006). The author reviews the concept of human societies adapting to global change from the perspective of adaptability and vulnerability and emphasizes that the adaptability and vulnerability of communities are closely related [39]. The most central is Fussel (2007). This article puts forward a conceptual framework of vulnerability that can be used in climate change, vulnerability, and adaptation assessment, which helps to build a bridge between climate change and vulnerability research methods [40].

The fourth largest cluster (#3) is labeled “Cotopaxi volcano”, and contains 29 articles with a silhouette value of 0.92. Most of the research results were published around 2009, focusing on social vulnerability and resilience in the context of natural disasters. The most active citation it contains is Marshall. The author assessed the social vulnerability of commercial fishermen and tourism operators to extreme weather events in coastal areas and proposed that enhancing adaptability is the focus of climate adaptation planning [41]. The most cited is Cutter (2008), who proposed the local disaster resilience model (DROP) and provided a set of candidate variables to implement the model, hoping to improve the comparative evaluation of disaster resilience at the local or community levels [42]. The most central is Frazier (2010), who examines the multiple stressors faced by land-use planning in coastal areas and proposes the need to explore adaptation strategies to deal with various disasters, providing an opportunity to integrate scientific knowledge on natural disasters and climate change with local social development concerns [43].

The fifth cluster (#4) is labeled “micro-level assessment” and contains 26 articles with a silhouette value of 0.98. It contains 11 citations, focusing on the testing and estimation methods for assessing social vulnerability, such as the robustness of indicator selection and the calculation of a social vulnerability index. The most active citation is Mafi-Gholami’s work. The author investigated the changes in social vulnerability as well as the changing socioeconomic conditions and environmental risks of counties in coastal counties over a 30-year period (1988–2017) and predicted social vulnerability in 2030, 2040, and 2050 according to climate change scenarios of drought intensity and sea level rise [44]. The research results of Tate (2012) have the highest citation frequency and centrality in this cluster and are an important knowledge base and knowledge turning point of cluster #4. Tate uses the global sensitivity and uncertainty analysis method to verify social vulnerability indicators under the influence of common natural disasters, evaluate the robustness of index rankings, and put forward specific suggestions for each stage of index construction [45].

## 4. Discussion

As one of the important research fields to promote sustainable social development, social vulnerability has been carried out in many pieces of research. However, there are still some research blind spots that have not been realized and need to be explored more comprehensively. Based on the above analysis, we present the prospects of future studies for social vulnerability, which include integrated and cross-scale research on social vulnerability as well as research on the occurrence and evolution, dynamic monitoring and optimal regulation of social vulnerability under multiple pressures.

### 4.1. Integrated Research on Social Vulnerability

Social vulnerability is the product of specific spatial, social, economic, demographic, cultural, and institutional environments and often shows a certain regional complexity and spatiotemporal dynamics due to differences in geographical location, base conditions, and external risks [7]. Through the analysis of cluster #0 and cluster #1, we found that the existing research is mostly stagnant in the assessment and static characteristic analysis of social vulnerability at the individual or family levels, and there are few studies on the spatiotemporal pattern and evolution process of social vulnerability. Although some studies have carried out the qualitative interpretation and quantitative identification of influencing factors, there is still a lack of in-depth exploration of the formation mechanism of social vulnerability. This situation may be limited by the difficulty, reliability, and integrity of acquiring long-term series data, resulting in social vulnerability researchers generally relying on questionnaire survey and in-depth interview data to carry out research. This is basically similar to the findings of Ran [1]. Ho also pointed out that the existing research is mostly isolated to studying the vulnerability of the spatial or temporal scales, and combining the spatial and temporal trends of social vulnerability can help to better adapt to thermal risks [46]. Therefore, under the complex background of global environmental change, advanced technologies such as 3S (GIS, GPS, and RS), big data, and spatial visualization should be utilized to focus on the integrated research on the patterns–processes mechanism in addition to the horizontal and vertical comparative research on social vulnerability. In addition, actively exploring regional development policies and risk aversion measures adapted to local conditions is crucial for the short-term and long-term adaptation and adjustment of social systems.

### 4.2. Cross-Scale Research on Social Vulnerability

The interaction of risks in different spatial and temporal scales often leads to a strong transmission effect of social vulnerability, and a change in a social system at a certain scale may lead to a change in a social system in a larger- or smaller-scale region [19]. However, in the analysis of cluster #2, we found that the existing studies were limited to single-scale vulnerability studies, such as on regions, cities, communities, and families, and there was a lack of cross-scale comprehensive research on social vulnerability such that it was difficult to comprehensively reflect the transmission characteristics and change trends, and the practical strategies of the research results could not be effectively implemented. In the future, it is necessary to focus on the changing characteristics, scale effects, transmission mechanisms, and cross-scale regulation strategies of social vulnerability between different scales so as to ensure that adaptation actions at different scales can be effectively implemented and achieve “multiplier” effects.

### 4.3. Research on Social Vulnerability under Multiple Pressures

Social systems are complex organisms often facing the influence of multiple pressures at the same time, which not only affect the risk management and adaptation decision making of human society but also cause social vulnerability in complex dynamic changes [47]. The impact of the interaction of multiple pressures on social systems is still a core issue of vulnerability science. From cluster #3, we found that existing studies pay more attention to the impact assessment of a single pressure on social systems, such as natural disasters, and there are few studies on social vulnerability under the common disturbances of nature and humanity. In particular, it is worth mentioning that the interaction of the global outbreak of COVID-19 and increasing climate change pressures has had a lasting impact on social systems. As emphasized by McCubbin et al., the vulnerability of water, land, livelihoods, and communities has increased significantly under the interaction of changing climate and non-climate pressures, and in the long-term, multiple stress factors must be considered in formulating adaptation measures [48]. In the future, on the basis of identifying the main multiple pressures faced by the study area, we should focus on the research on the occurrence process and evolution law of social vulnerability in multiple-stress environments. Revealing the multiple-feedback relationship between multiple pressures and social system elements, this is not only an important way to correctly understand the process of social development but also helps to promote human beings to take effective adaptive actions to achieve social sustainable development. In addition, integrated research on social vulnerability and adaptation provides a new paradigm for promoting social sustainable development. In the future, it is necessary to consider the trade-off and selection of adaptation modes, the identification of adaptation barriers, the evaluation of adaptation effects, and other related issues in the research on social vulnerability.

### 4.4. Research on the Dynamic Monitoring and Optimal Regulation of Social Vulnerability

The combined effect of risk and social vulnerability will lead to the eventual transformation of risks into disasters, which is mainly due to the vulnerability attribute of social systems [49,50]. In view of the complexity of social systems and the uncertainty of risk, the establishment of social vulnerability monitoring mechanisms and risk control mechanisms is helpful to reduce the impact of disasters from the source. Based on the analysis of high-frequency keywords, emergent keywords, and high-centrality keywords, we found that keywords such as climate change, risk, disaster, perception, risk assessment, impact, and adaptation appear repeatedly, indicating that risk research is still the focus in the field of social vulnerability. At present, some scholars have set up climate change scenarios to predict the degree of social vulnerability in a certain period in the future, such as the results of the most active references [44] in cluster #4. However, current prediction studies are mostly limited to the impact of future risks on the current social systems. Such time dislocation may lead to incorrect estimations of future social vulnerability, which is consistent with the conclusion of Hardy [51]. In the future, comprehensive research should be carried out based on multisource datasets and by using OWA algorithm, system dynamics, and social network analyses as well as other interdisciplinary methods. It should focus on the change characteristics, occurrence mechanisms, regulation strategies, and synergistic effects of social vulnerability under multi-scenario simulations. Theoretical aspects can deeply grasp the development trend of social vulnerability. Practical aspects not only help to implement targeted adaptation strategies in current vulnerable areas but also help areas where vulnerability may increase in the future to adopt targeted prevention and control strategies.

## 5. Conclusions

Research on social vulnerability is helpful to seek effective countermeasures to reduce the adverse impacts of environmental change and human activities on social systems. This study systematically reviewed 2710 articles by using CiteSpace and explored the knowledge structure and new trends in the field of social vulnerability research through a co-occurrence visualization map of countries, institutions, keywords, and documents. On the one hand, this research can enrich the research scope of social vulnerability and help monitor the research development of this topic, investigate its research changes, and predict future research trends. On the other hand, this study provides an overall overview of the knowledge structure for researchers, especially students and novices in this field, which is of great significance for theoretical research on and the practical exploration of social vulnerability.

This study found that, from 1990 to 2021, the number of studies on social vulnerability research showed a steady upward trend and experience roughly three stages: an embryonic stage, a development stage, and a stable stage. From the perspective of the main contributing countries and institutions of social vulnerability research, the United States, Brazil, the United Kingdom, and China have widely established the discipline system of social vulnerability research. We found that the main contributors to social vulnerability research are universities in these countries, and they have established domestic and international cooperation networks within and between them. While these institutions published a large number of research articles on social vulnerability, the nodes representing them also had high centrality and emergence, with half-lives of more than 10 years, indicating that these institutions were very active in their research activities. Nevertheless, the cooperation intensity between these countries or universities is still weak, and it is still necessary to strengthen academic exchanges and cooperation in the future. It is worth noting that while social vulnerability is generally higher in developing countries or regions, there are more studies from countries such as the United States than from any other country. The United States and the United Kingdom conducted research on social vulnerability almost simultaneously, but the United States published 4.7 times more research than the United Kingdom did. From the perspective of keyword co-occurrence visualization, “climate change”, “vulnerability”, “risk”, “resilience”, “impact”, “hazard”, and “adaption” were the main keywords of social vulnerability research published over the past 32 years. At present, research on social vulnerability to environmental stresses such as climate change and natural disasters is dominant, accounting for about 51% of the 2710 articles that we systematically reviewed. This is similar to the study by Fatemi et al., who selected 43 out of 137 articles to review the effectiveness of social vulnerability indices in disasters and found that 83% of the studies were related to different types of natural disasters [52]. As we found in the previous analysis of keywords in different years, only 24 high-frequency keywords appeared from 1990 to 2005, and the research content was relatively singular. From 2006 to 2021, 120 high-frequency keywords appeared, showing a trend of diversification. With the diversification and deepening of keywords, social vulnerability research under the background of social and economic activities such as urbanization and tourism development has begun to attract attention. In addition, five typical clusters are identified according to the cited publications, and they can be divided into five topics: social vulnerability studies on individuals or families and other groups, the evaluation and driving factors of social vulnerability, vulnerability and adaptability research of multiregional levels or multiple risks, social vulnerability and resilience under natural disasters, and testing as well as estimation methods for assessing social vulnerability. Finally, based on the results of a bibliometric analysis, we put forward the topics that should be paid more attention to in the future. They mainly include integrated and cross-scale research on social vulnerability, research on occurrence its evolution, and the dynamic monitoring as well as optimal regulation of social vulnerability under multiple pressures.

In summary, we used CiteSpace to conduct a comprehensive analysis of the social vulnerability research carried out worldwide from 1990 to 2021. The analysis of each feature can show different aspects in the field of the topic. The increasing trend of social vulnerability research can be summarized by the number of published studies and its annual growth rate. Carrying out analyses on contributing countries/regions and institutions can identify representative countries and key institutions of social vulnerability research, which can provide a basis for relevant scholars to continue their research and find partners. Keyword co-occurrence and emergence can capture the research hotspots and internal relations in the field of social vulnerability. A cluster analysis of publication co-citations can determine representative studies and research frontiers in the field of social vulnerability in addition to learning about clustering topics, research contents, and other information of existing studies. Although we have fully considered the major databases included in the *WOS* collection, publications in this area may be insufficient. For example, search subject scope, document type limitations, and differences in language coverage can pose thorny issues for bibliometrics. In the future, it is necessary to comprehensively consider multiple database document types and different languages to explore research hotspots and development trends of social vulnerability in a more systematic and comprehensive way.

## Figures and Tables

**Table 1:** The five largest clusters sorted by size.

Cluster ID	Size	Silhouette	Mean Year	Label (LLR)	The Most Actively Cited Publication	Publications Cited with Highest Frequency	Highest Centrality Publications	Burst
0	38	0.81	2009	Private household	Tate [33]	Hinkel [34]	Fekete [35]	15
1	31	0.97	2015	Social vulnerability	Ran [1]	Rufat [36]	Armas [37]	6
2	30	0.73	2006	Multiple level	Bisaro [38]	Smit [39]	Füssel [40]	7
3	29	0.92	2009	Cotopaxi volcano	Marshall [41]	Cutter [42]	Frazier [43]	3
4	26	0.98	2012	Micro-level assessment	Mafi-Gholami [44]	Tate [45]	Tate [45]	11

## Data Availability

Not applicable.
